# Pulmonary Fissure Segmentation in CT Images Using Image Filtering and Machine Learning

**DOI:** 10.3390/tomography10100121

**Published:** 2024-10-09

**Authors:** Mikhail Fufin, Vladimir Makarov, Vadim I. Alfimov, Vladislav V. Ananev, Anna Ananeva

**Affiliations:** Medical Informatics Laboratory, Yaroslav-the-Wise Novgorod State University, 41 B. St. Petersburgskaya, Veliky Novgorod 173003, Russia; vladimir.makarov@novsu.ru (V.M.); vadim.alfimov@novsu.ru (V.I.A.); vladislav.ananev@novsu.ru (V.V.A.); s235373@std.novsu.ru (A.A.)

**Keywords:** lung, fissure, segmentation, computed tomography, machine learning, CNN, stick derivative

## Abstract

Background: Both lung lobe segmentation and lung fissure segmentation are useful in the clinical diagnosis and evaluation of lung disease. It is often of clinical interest to quantify each lobe separately because many diseases are associated with specific lobes. Fissure segmentation is important for a significant proportion of lung lobe segmentation methods, as well as for assessing fissure completeness, since there is an increasing requirement for the quantification of fissure integrity. Methods: We propose a method for the fully automatic segmentation of pulmonary fissures on lung computed tomography (CT) based on U-Net and PAN models using a Derivative of Stick (DoS) filter for data preprocessing. Model ensembling is also used to improve prediction accuracy. Results: Our method achieved an F1 score of 0.916 for right-lung fissures and 0.933 for left-lung fissures, which are significantly higher than the standalone DoS results (0.724 and 0.666, respectively). We also performed lung lobe segmentation using fissure segmentation. The lobe segmentation algorithm shows results close to those of state-of-the-art methods, with an average Dice score of 0.989. Conclusions: The proposed method segments pulmonary fissures efficiently and have low memory requirements, which makes it suitable for further research in this field involving rapid experimentation.

## 1. Introduction

The human lungs are divided by fissures into anatomically independent lobes. In clinical practice, the segmentation of lung lobes is useful for the diagnosis and evaluation of lung diseases [1]. It is often of clinical interest to quantify each lobe separately because many diseases are associated with specific lobes. For example, pulmonary lobe segmentation methods can be used to assess the infection severity of COVID-19 by individual lobe [2]. In addition, the segmentation of lobes is extremely important in the surgical treatment of lung diseases [3,4]. For example, the surgical treatment of non-small cell lung cancer often includes lobectomy [5], which is the removal of the diseased lobe.

Therefore, analyzing an affected lung region at the individual lobe level can provide valuable insights for the purpose of the diagnosis and evaluation of a variety of medical conditions.

One of the most popular approaches to lobe segmentation estimates lobar boundaries based on information from fissures, airways, and vessels [6,7,8]. In addition, there is an increasing requirement for fissure integrity or completeness quantification, which is highly relevant to lung disease characterization [9]. Some studies have proposed methods for the automated analysis of pulmonary fissure integrity [10,11].

Fissure segmentation is important for a significant proportion of lung lobe segmentation methods as well as for assessing fissure integrity. However, there are a lot of challenges that make automatic fissure segmentation a difficult task, namely, imperfections in computed tomography (CT) technology, the presence of image noise, inhomogeneous image intensity, natural variability in lung anatomy, and variability in lobe shape due to the influence of lung diseases.

A fissure is a double layer of connective tissue formed by invagination of the outer pleural membrane of the lung [12]. The left lung consists of two lobes separated by a left oblique fissure. The right lung consists of three lobes separated by a right oblique fissure and a right horizontal fissure.

The lobe boundaries formed by pulmonary fissures are often either partially invisible on CT images or difficult to distinguish from the adjacent vessels, bronchi, and pathological structures. On cross-sectional CT images, fissures show up as thin curve-like structures (less than 1 mm thick), slightly denser than the surrounding lung parenchyma. Often, fissures are only partially visible or even absent on one or more cross-sections. Moreover, due to lung diseases, serious deformations of the shape of individual lobes can happen. Pathologies such as fibrosis or emphysema may locally resemble fissures or obstruct their shape and appearance [13].

Even CT scans of healthy patients show high anatomical variability, which in itself poses a great challenge in the task of segmenting pulmonary fissures and/or lobes and requires consideration of the larger context surrounding the object of interest. Another factor to consider is the quality of the image. Fissures can be indistinguishable on low-resolution CT scans (with slice thickness greater than 1 mm) so that even a human cannot recognize them clearly. In this case, even creating a dataset to train or validate the algorithm can be difficult, because the worse the image quality, the more disagreement between the experts. The presence of noise has similar issues to low resolution.

The accurate segmentation of lobes requires both local and global information to be taken into account. Local information here means the intensity of voxels located in the vicinity of the fissure within a radius of at least one order of magnitude less than the image size. The presence of vessels, bronchi, airways, and ribs of the thorax is an example of global information, which helps in narrowing down the fissure detection area. This information is especially useful when a fissure in the image does not have enough contrast, is missing, or looks sparse. Because of these issues, local information is not sufficient to identify fissures and/or lung lobes with enough confidence.

Convolutional neural networks (CNNs), as a class of deep learning (DL) models, are effective in many computer vision tasks. They can extract complex and non-obvious correlations from data without relying on models invented for the specific problem. Instead, each model architecture typically solves a broad class of problems, and for a particular problem, it is trained on domain-specific data in a process known as machine learning (ML). Deep learning is a type of machine learning that involves training neural networks with hidden layers, such as convolutional neural networks. Since we do not use any other type of ML in this paper, hereafter, the terms ML and DL will be used interchangeably.

ML is applied for tasks like fissure and lung lobe segmentation as well. Depending on the particular kind of neural network architecture applied in the machine learning model, either individual CT slices or 3D segments may be used as its input. Based on the input type, fissure and lobe segmentation methods can be divided into volumetric and non-volumetric. The advantages of non-volumetric segmentation neural networks are low memory consumption and lower dataset size requirements. The advantage of volumetric neural networks is that such models do not lose relationships between slices, as the input is a 3D image rather than separate slices. This allows for performing fissure and/or lobe segmentation even when the fissure cannot be recognized on some cross-sections.

Before the widespread adoption of neural networks, non-ML fissure and lobe segmentation algorithms served as the basic approach to the task. Their advantage is that they require much less data for the parameter search and validation.

Early attempts to segment pulmonary fissures and lobes were based on various methods, such as the watershed transform [14,15], Voronoi division [16], adaptive sweeping [17], and minimal path [18]. Many of them were based on the Derivative of Stick (DoS) filter proposed by Xiao et al. [6]. The DoS filter enhances fissures in the scan by processing individual slices of a certain cross-section. Filtering is performed in three cross-sections orthogonal to each other, and then the results are combined. Next, thresholding is performed using multiple thresholds, and these results are also combined. To eliminate false positives, a postprocessing pipeline based on a 3D connected component analysis is used. Based on this work, Peng et al. [7] introduced a new framework based on lung anatomy knowledge and airway and pulmonary fissure segmentation using ODoS [19], an improved version of the DoS method, to segment lobes. Zhao et al. [20] proposed an anisotropic differential operator called the directional derivative of plate (DDoP) filter, which is a 3D version of the DoS filter. Chen et al. [21] segmented pulmonary lobes by applying a multistage spline surface fitting method to the masks obtained with the DDoP filter. Ross et al. [22] also used a thin-plate spline [23] surface fitting method to segment pulmonary lobes with lobar fissure masks as input data, although they employed a particle system rather than a DoS-based algorithm to segment fissures.

Non-ML algorithms are less efficient in taking global information into account and are more sensitive to changes in data sampling. It is an extremely difficult task to devise an algorithm that utilizes enough global information to produce reliable results. The reason is that the fissure position and shape depend on global information in a non-obvious way due to natural anatomic variability. Because of this, many modern approaches to this problem make use of CNNs.

For example, Gerard et al. [24] used a model called FissureNet that is composed of two Seg3DNet networks. One Seg3DNet model finds the region of interest around pulmonary fissures, and another model of the same type refines the prediction of the previous network. FissureNet is trained separately for each lung, with CT scans split into 3D chunks of 64 × 200 × 200 voxels. Gerard and Reinhardt [25] proposed the LobeNet model, which is a FissureNet extension for lobe segmentation. LobeNet consists of four Seg3DNet networks. The first two networks correspond to the two Seg3DNet networks of FissureNet and segment pulmonary fissures. The third Seg3DNet network uses CT scan data and lung fissure segmentation masks to coarsely estimate lobe boundaries. The fourth network refines the results of the previous one. Later, Gerard, Herrmann, et al. [26] used LobeNet to develop a segmentation algorithm that predicts left and right lung regions in humans with diffuse opacification and consolidation.

As ML methods have evolved, an increasing number of authors have proposed approaches that segment lung lobes using grayscale information directly, avoiding the fissure segmentation step. Park et al. [27] used 3D U-Net to segment pulmonary lobes on CT scans. Wang et al. [28] used V-Net for the same segmentation task with 3D chunks of CT scans as input. To better account for global and positional information, Wang et al. [28] added a CoordConv layer to the network. CoordConv is a simple extension of a regular convolutional layer to incorporate positional information by including additional channels for voxel coordinates.

Some researchers have chosen non-volumetric CNNs for pulmonary fissure and lobe segmentation tasks. Chen et al. [29] proposed a scheme called LLASN (Lung Lobes Adversarial based Segmentation Network) in which U-Net is used to generate segmentation results, and a discriminator network is used to discriminate the generated segmentation results from ground-truth labels. Dadras et al. [30] employed multiple ML techniques (self-supervision, attention, and augmentation) to train a lung lobe segmentation model based on 2D U-Net.

We propose a method for the fully automatic segmentation of pulmonary fissures on lung CT based on a DoS filter and non-volumetric CNNs. The advantage of our method over ordinary segmentation networks working with individual slices is better performance due to adding a DoS filter as a preprocessing step to account for the volumetric information of the input image. Model ensembling is also used to improve prediction accuracy.

The advantage of the proposed method over methods based on volumetric CNNs such as 3D U-Net and V-Net is that it requires less memory. This can be easily demonstrated by the fact that a single slice of a 3D image is an edge case of a 3D chunk, and while non-volumetric CNNs can be trained on batches as small as a singular slice, volumetric CNNs should use wider chunks to have a measurable advantage over non-volumetric CNNs.

The main contributions of this paper are the following:

- This paper proposes a novel method for pulmonary fissure segmentation on lung CT using 2D CNNs and the DoS filter.
- We suggest a pulmonary lobe segmentation method using a fissure detection algorithm and an interpolation technique known as thin-plate splines.
- We draw more attention to the problem of the automatic segmentation of objects such as parenchyma, fissures, lobes, vessels, and airways, which can be helpful in diagnosis and surgical planning.

This paper is structured as follows. Section 2 describes the segmentation pipeline, including model training, training loss design, and the DoS filter used to preprocess data for model training and validation, as well as for inference. This section also provides a classification of the segmentation errors used in this paper and describes the data used for the experiments. Section 3 shows the experimental results, including a comparison of the proposed method with the DoS method, cross-validation results, and lobe segmentation experiment results. In Section 4, we evaluate the performance of the proposed method, discuss its advantages as well as limitations and weaknesses, and suggest how the proposed approach can be improved and/or be utilized in the future.

## 2. Materials and Methods

### 2.1. Derivative-of-Stick Filter

The key idea of the DoS filter is to use stick filters of L×L size, where *L* is the length of the stick. The image is filtered with 2(L−1) versions of the filter, one for each possible filter orientation. The lung parenchyma tissue has the lowest density in areas immediately adjacent to the fissures. To take advantage of that, Xiao et al. [6] use three parallel sticks spaced *S* pixels apart from each other instead of a single stick.

Two nonlinear derivatives for fissure enhancement are introduced, $\ell_{\max}$ and $\ell_{\min}$:

(1) $$\ell_{\max}^{S,\theta }\left(x\right)=\max\left(\mu_{M}-\mu_{L},\mu_{M}-\mu_{R}\right)-\kappa \cdot \sqrt{E\left[I_{j}^{2}\right]-{\left(E\left[I_{j}\right]\right)}^{2}},$$

(2) $$\ell_{\min}^{S,\theta }\left(x\right)=\min\left(\mu_{M}-\mu_{L},\mu_{M}-\mu_{R}\right)-\kappa \cdot \sqrt{E\left[I_{j}^{2}\right]-{\left(E\left[I_{j}\right]\right)}^{2}}.$$

Here, θ is the orientation angle of the stick, *x* is the spatial position, and κ is a positive coefficient.

$\mu_{M}$, $\mu_{L}$, and $\mu_{R}$ are the mean intensity values along the middle, left, and right sticks, respectively:

(3) $$\mu =\frac{1}{L}\sum_{j=1}^{L}I_{j}.$$

Here, *E* is the expected value operator, and $I_{j}$ is the intensity of the *j*-th pixel. The second term is introduced to suppress blob-like structures.

The stick responses of different orientations can be integrated as follows:

(4) $$F_{\max}\left(x\right)=\max\left(\max_{1\leq i\leq 2(L-1)}\left(\ell_{\max}^{S,\theta_{i}}\right),\;0\right)$$

(5) $$F_{\min}\left(x\right)=\max\left(\max_{1\leq i\leq 2(L-1)}\left(\ell_{\min}^{S,\theta_{i}}\right),\;0\right)$$

Here, $\theta_{i}$ denotes the discrete angle of the *i*-th stick. Since we are looking for bright objects, only non-negative response values are considered.

A fissure with a step-like appearance or a thickened fissure can be converted into a standard thin curvilinear structure using the $F_{max}$ operation. Normal thin fissures are not affected by $F_{max}$. When $F_{min}$ is applied to the result of $F_{max}$, both normal and pathological fissures will be enhanced equally. The combined filter can be described as follows:

(6) $$F_{\circ }=F_{\max}\circ F_{\min}.$$

Because a fissure can be barely visible or interrupted on slices in one cross-section and clearly visible in another, Xiao et al. [6] integrate responses from all three perpendicular cross-sections:

(7) $$F^{3D}\left(x\right)=\left(F_{\circ }^{A}+F_{\circ }^{S}+F_{\circ }^{C}\right)\cdot \frac{\mathrm{median}\left(F_{\circ }^{A},F_{\circ }^{S},F_{\circ }^{C}\right)}{\max\left(F_{\circ }^{A},F_{\circ }^{S},F_{\circ }^{C}\right)}.$$

Here, $F_{\circ }^{A}$, $F_{\circ }^{S}$, and $F_{\circ }^{C}$ denote the response of the DoS $F_{\circ }$ filter in the axial, sagittal, and coronal cross-sections, respectively.

### 2.2. The Proposed Method

We introduce two new segmentation methods in addition to the baseline method, in which a neural network is applied without preprocessing data with a DoS filter. By comparing different methods, we tried to answer two questions: which cross-section gives the best result and whether preprocessing a CT scan with a DoS filter improves the result. To answer these questions, three image preprocessing approaches were chosen. The first performs histogram equalization and intensity normalization to the 0…255 range. The second also involves histogram equalization and normalization, but the image is first processed by a DoS filter. The third approach combines the outputs of the previous two methods by creating an additional data dimension.

**Figure 1:**
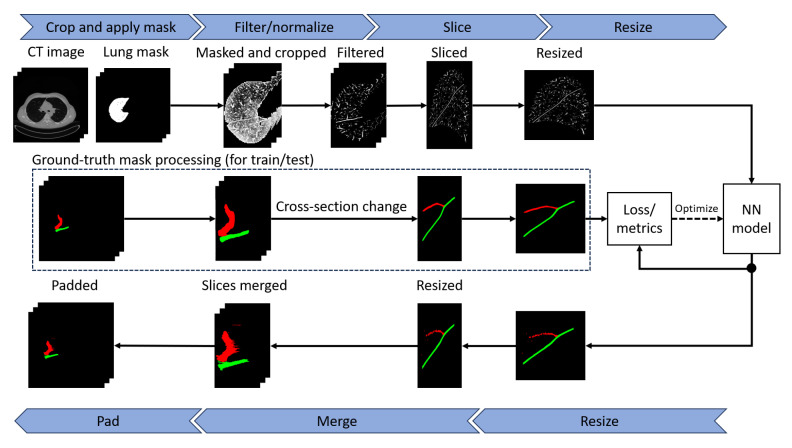
The segmentation pipeline overview. First, the image is masked with the lung mask and then cropped to the mask’s bounding box. Next, the image is normalized and optionally filtered by a DoS filter. Then, individual slices are fed as input to the neural network. Finally, several inverse operations are performed to transform the output and retrieve the fissure segmentation. Green and red colors indicate voxel values that depend on the object class, and black is the background.

The pipeline of the image segmentation application is shown in Figure 1. First, a lung mask is applied to the image in such a way that everything outside the mask is filled with zeros. The image is also cropped to a region of interest that is equal to the bounding box of the lung mask. Then, the image is filtered and/or normalized depending on the preprocessing approach of choice. The 3D scan is then sliced. As the previous step produces images of variable size due to the natural variability in lung anatomy and differences in CT scan resolutions, slices are resized to 512×512 pixels. This step is performed because the model deals with images of the same size.

The segmentation model expects individual slices as input. The neural network outputs a mask with integer values from 0 to 2, where 0 is the background, 1 is an oblique fissure, and 2 is a horizontal fissure. All of the preprocessing steps, except for DoS filtering, have an inverse postprocessing step. The mask then gets scaled back to the cropped image resolution. The individual slices are merged into a 3D image, which is then padded to the size of the original CT image.

**Figure 2:**
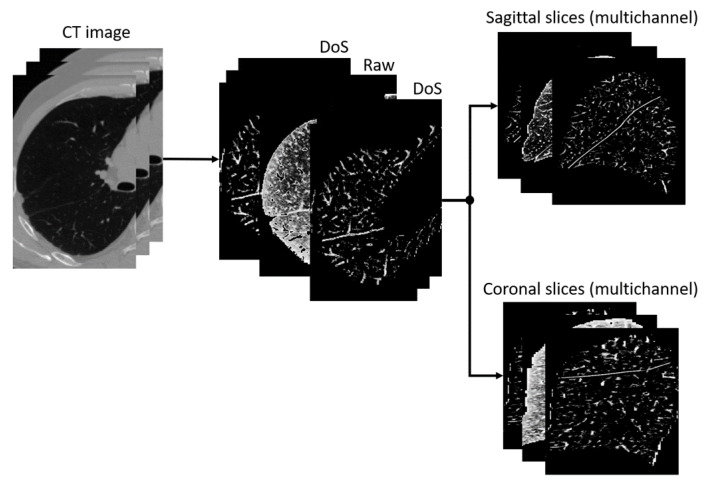
The combined preprocessing approach pipeline. Only one image channel contains the normalized image not processed by the filter.

Figure 2 depicts a pipeline of the combined preprocessing approach. Firstly, the image is processed with the DoS filter, and then histogram equalization and normalization to 0…255 are applied. The output image has three channels of the same size as the input. All channels except for the second contain the same image processed by the DoS filter. The second channel contains the image prepared in a similar way except for the DoS filtration. The result is then sliced and saved into separate images for further use in model training and testing.

Six models were trained for each lung, one for each combination of preprocessing approach and cross-section (either sagittal or coronal). There are twelve models in total, not including the standalone DoS method, ensembles, and the models trained for the cross-validation. For two of them, PAN architecture and the Focal loss were used, and for the rest, U-Net and the Dice loss were used, which is explained in Section 3. The axial cross-section is not used because the horizontal fissure of the right lung appears only on a few slices due to its orientation (hence its name). Therefore, a training dataset composed only of axial slices would be very unbalanced. Though usually an issue like that is solved by applying augmentations, there is no clear way to solve this issue in this particular case.

**Figure 3:**
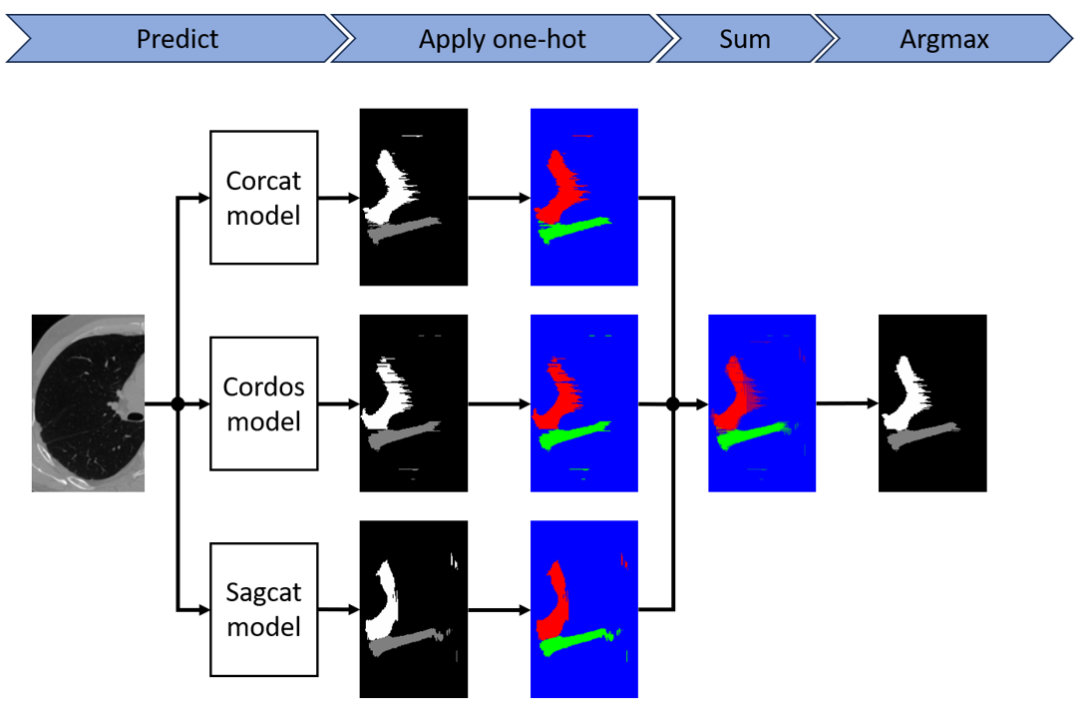
Model ensembling pipeline. One-hot is applied to the output of each model, and then argmax is applied to the sum of the results. Here, black-and-white images represent the standard coding of classes, where for each class a particular value is used. Here, black and white images represent standard class encoding, where a specific value is used for each class. Color images represent one-hot class encoding, each color refers to one of the channels (blue—background, red and green—fissures).

To further improve the performance of the models, we apply model ensembling. The pipeline is shown in Figure 3. The one-hot function is applied to each of the segmentation masks generated by the models that make up the ensemble, and then the argmax function is applied to the sum of the results. To ensure the reliability of the results, we also performed 10-fold cross-validation.

### 2.3. Error Types

**Figure 4:**
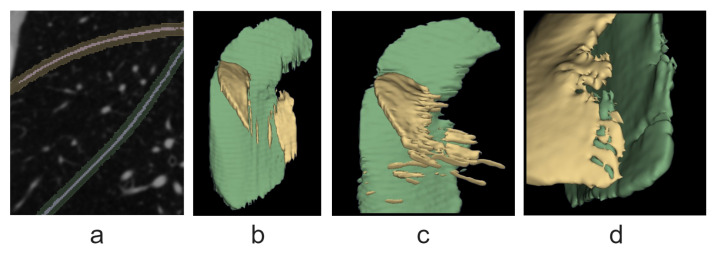
Error types shown in the right lung segmentation example: (**a**) oversegmentation; (**b**) false negatives; (**c**) false segmentation; (**d**) misclassification. Green indicates the oblique fissure and yellow indicates the horizontal fissure.

Fissure segmentation errors can be divided into the following categories: false negatives (type II errors), misclassification, oversegmentation, and false fissure segmentation (or simply false segmentation). The last two are examples of false positives (or type I errors). Examples are shown in Figure 4. Oversegmentation (see *a*) occurs when the predicted mask overlaps with the true mask along the entire fissure cross-section, but the Jaccard measure (the overlap area divided by the union area) is less than 70%. False segmentation (see *c*) is the incorrect classification of structures’ voxels as fissures. Misclassification (see *d*) happens when the model attributes a voxel to the wrong fissure type.

There are methods to mitigate errors of each type. The postprocessing stage of the DoS method is an example of a false positive reduction algorithm. In this paper, we use model ensembling to eliminate false segmentation and to increase the overall accuracy of the segmentation as well.

To reduce the misclassification error rate, we propose an algorithm based on the *k*-nearest neighbor (KNN) approach. The idea is to keep the two largest components, one for each of the two classes (for the right lung), and train the KNN method on the coordinates of these voxels. This allows falsely classified voxels to be assigned to a more appropriate class based on their proximity to a particular fissure. For this purpose, all voxel coordinates of the original mask are passed to the trained model, resulting in a new class being assigned to them. Although an ensemble model is capable of reducing a large proportion of such errors by itself, curvilinear approximation algorithms that derive the lobe segmentation are very sensitive to misclassification. Therefore, the proposed algorithm can be used as a precautionary measure.

Oversegmentation errors are usually not critical, because in both the predicted segmentation and the true segmentation, the fissure masks are wider than real fissures, which look like very thin lines in sagittal and coronal sections. Since we consider fissure segmentation as an intermediate step in the lobe segmentation task, usually followed by curvilinear approximation (e.g., using the thin-plate spline method [23]) or an additional lobe segmentation model [25], the mask width does not play a major role as long as it does not interfere with the approximation step.

Moreover, oversegmentation is preferable to type II errors or false segmentation because people usually highlight not the fissure itself but the approximate area around it, and the width varies not only in annotations made by different people but also in annotations made by the same person. But, since standard measures such as $F_{1}$, the Jaccard coefficient, and the Dice coefficient do not take this into account, they encourage accuracy instead of completeness.

The simplest solution would be to use the measures that allow for configuring the importance of false positives over false negatives, such as $F_{beta}$. However, such measures would equally encourage oversegmentation and false segmentation. It is possible to modify a measure so that oversegmentation will have less effect on its value than false segmentation. In this paper, we use the measures proposed by Xiao et al. [6], which satisfy this requirement.

The changed Precision, Recall, and $F_{1}$ measures are based on counting true positives as well as type I and type II errors. Xiao et al. [6] use a 3 mm margin to define true positive values: i.e., voxels that are no more than 3 mm away from the ground-truth mask are considered true positive values for $TP_{1}$, and all other voxels of the predicted mask contribute to type I errors or false positives FP [6]. In a similar way, the voxels of the ground-truth mask are divided into $TP_{2}$ and type II errors FN, for which the same intersection criterion is used. In general, $TP_{1}$ and $TP_{2}$ are not equal. Then, Precision and Recall measures are defined as $TP_{1}/\left(TP_{1}+FP\right)$ and $TP_{2}/\left(TP_{2}+FN\right)$. The $F_{1}$ measure is defined by the same equation: $F_{1}=2\cdot Precision\cdot Recall/\left(Precision+Recall\right)$.

### 2.4. Loss Function

In this paper, we use both the Dice loss function [31] and the Focal loss function. Both the Dice loss and Focal loss perform well in tasks with high class imbalance, like fissure segmentation. The Dice loss is based on the Dice score, while the Focal loss is an improved version of the Cross-Entropy loss. The Focal loss has been described in detail in [32]. The Dice loss function is defined as follows:

(8) $$D=\sum_{c}D^{c}=\sum_{c}\left(-\frac{2\sum_{i}p^{c}\left(i\right)g^{c}\left(i\right)}{\sum_{i}p^{c}\left(i\right)+\sum_{i}g^{c}\left(i\right)+\gamma }\right)$$

where $p^{c}\left(i\right)$ and $g^{c}\left(i\right)$ represent the predicted value and ground truth at position *i*, respectively; *c* denotes the class number; and γ is a very small positive value (e.g., $1\times 10^{-5}$) to avoid division by 0.

### 2.5. Data

Ninety-nine chest CT scans were collected from the Novgorod Regional Clinical Hospital. The patients who had undergone the scans had been diagnosed with lung cancer or had symptoms of lung cancer. The scans were picked according to the following criterion: the pulmonary fissures are clearly visible to a physician over most of the area of the respective lung.

Scans were produced by different scanners, namely, Aquilion manufactured by Toshiba in Shimoishigami, Otawara-shi, Tochigi, Japan, Ingenuity manufactured by Philips Healthcare in Cleveland, Ohio, USA, Optima CT660 manufactured by GE BE Private in Whitefield, Bangalore, India, BrightSpeed manufactured by GE Hangwei Medical Systems in Beijing, China, as well as SOMATOM Definition AS, Sensation, Perspective, and Emotion all manufactured by Siemens in Erlangen, Germany. The slice thickness ranged from 0.3 mm to 2 mm, with 86 out of 99 scans (87%) having a slice thickness of 1 mm or less.

Our access to the data was facilitated through the Cooperation Agreement between the Ministry of Health of the Novgorod Region and the Novgorod State University (Agreement number 20230609, signed on 9 June 2023). The study was conducted in accordance with the Declaration of Helsinki, and protocol #5 was approved by the Novgorod State University Ethics Board (approval date: 22 January 2024).

Nine scans were randomly selected for the test set, and the remaining ninety scans were used as training data. Pulmonary fissures on the scans were manually annotated by a physician using 3D Slicer [33]. Lung masks were obtained automatically using the Chest Imaging Platform software (version 5.2.2) based on 3D Slicer (version 5.5.0-2023-11-24) [34].

## 3. Results

### 3.1. Fissure Segmentation

The segmentation models were trained using Pytorch version 2.1.0 on an NVIDIA Quadro RTX 8000 graphics card with 48 GB of memory. The following parameters were used for the DoS filter: L=11, gap=3, and k=0.5. The values of *L* and gap were taken from [6], and the value k=0.5 was chosen experimentally. The rotate, translate, scale, and shear augmentations from the Albumentations package [35] were used in training. The U-Net and Pyramid Attention Network (PAN) architectures, along with the Focal and Dice loss functions, were used. The specific implementations of these architectures and loss functions were taken from the Segmentation Models Pytorch Python package [36].

There are two models per data preprocessing method, one trained on the slices obtained in the sagittal cross-section and one trained on the coronal slices, which gives us six models for each lung and twelve models in total. For brevity, the models were given the following designations. Models trained on sagittal slices have the prefix *sag*, and models trained on coronal slices have the prefix *cor*. Models trained on data preprocessed by the DoS filter have the suffix *dos*. Models trained on data not processed by the filter have the suffix *raw*. Models working on hybrid-type data (as shown in Figure 2) have the prefix *cat* (short for concatenation, since it concatenates the results of the other two approaches). The names of the models included in the ensemble are abbreviated to the first letters of the prefix and suffix (e.g., *cd* is the same as *cordos*, i.e., the model trained on coronal slices using the DoS filter). The abbreviation reference can be found in Table 1.

The combination of the U-Net architecture, the ResNet 34 encoder, and the Dice loss function was used as the baseline method. The PAN and DeepLabV3+ models with the ResNet 101, ResNet 152, EfficientNet B3, and EfficientNet B4 encoders were also tested. With the exception of the *sagcat* models (hybrid processing, sagittal cross-section), no significant improvement over the baseline was observed during the validation phase. For all other models, the baseline was chosen. The *sagcat* models are PANs with the ResNet 34 encoder and were trained using the Focal loss function.

For the right lung, the models were trained on slices of 90 volumetric CT images, and test data consisting of slices of 9 CT images were used for evaluation. The resulting comparison of the models with one of the ensembles is shown in Table 2. The ensemble schematic can be seen in Figure 3. In addition to the Precision, Recall, and $F_{1}$ scores, their alternative versions, described earlier in this paper and named $P_{DoS}$, $R_{DoS}$, and $F_{DoS}$, were also used. These scores are less sensitive to oversegmentation than the standard Precision, Recall, and $F_{1}$ scores. With the exception of Recall and $R_{DoS}$, the ensemble consistently performs better in all other measures, since ensembles tend to sacrifice Recall for Precision.

**Figure 5:**
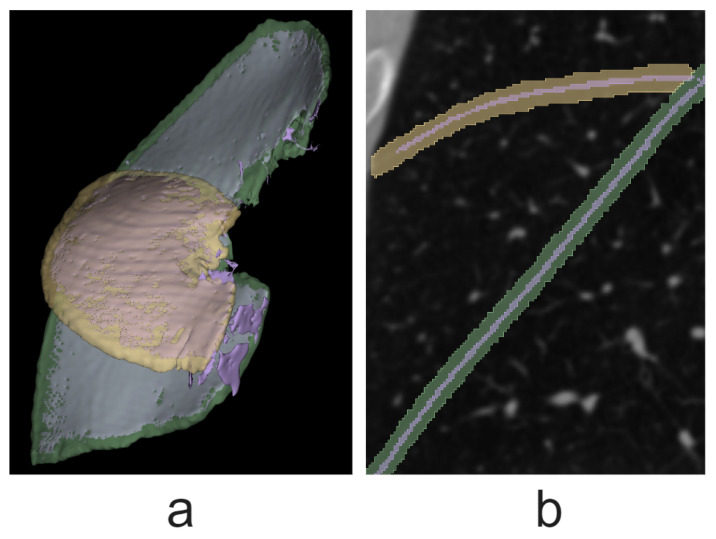
A comparison of the ensemble of neural network models and the DoS method using one of the test images. The mask obtained with DoS is highlighted in purple. $F_{DoS}=0.908$ for the DoS result, and $F_{DoS}=0.967$ for the ensemble result (measured on this scan alone). (**a**) A 3D model. (**b**) A sagittal slice.

The comparison of the six-model ensemble with the original DoS algorithm of [6], which includes the 3D connected component analysis pipeline, is shown in Table 3. The visual comparison of the masks obtained using the ensemble and using DoS is shown in Figure 5. Since DoS does not distinguish between oblique and horizontal fissures of the right lung, all fissures had the same class in the comparison. We can conclude that the DoS algorithm is much less effective than neural network-based methods.

For both the right and left lungs, we combined the 6 models to create 57 ensembles. The top five models for the right lung based on the $F_{DoS}$ score are shown in Table 4.

The $F_{1}$ and $F_{DoS}$ scores for each of the right-lung fissures are presented in Table 5.

For the left-lung fissure segmentation, the same approach was used as for the right-lung fissure segmentation. Specifically, models were trained on slices obtained in two cross-sections with different preprocessing techniques (L-models), and then model ensembles were created. A training set of 68 images and a test set of 9 images were used for the left-lung fissure segmentation, with encoders’ weights initialized with the weights from the respective right-lung fissure segmentation model encoders before training. For example, the left-lung *sagcat* model encoder weights were initialized with the weights of the same model of the right lung. All but the largest connected component of the oblique fissure were also removed before testing.

Table 6 shows the comparison of the best single model, the two best ensembles, and the standalone DoS method. We also used models trained for right-lung fissure segmentation (R-models) to segment left-lung fissures. The comparison results can be seen in the same table, namely, the best single model and the best ensemble. In the task of left-lung fissure segmentation, the $F_{DoS}$ of R-models is just 2.6% lower than that of L-models, which shows that R-models have achieved significant generalization ability.

### 3.2. Cross-Validating Fissure Segmentation Models

Since the training and test datasets we used for training and evaluation are relatively small, we performed 10-fold cross-validation to ensure that the results are reliable. For this purpose, we combined the training and test datasets into two sets of 99 and 77 scans each for the right and left lungs, respectively. We then divided the first set into 10 folds of 9 or 10 CT scans and the second set into 10 folds of 7 or 8 scans for the left lung. The second-best ensemble for the right lung and the second-best ensemble for the left lung were also re-evaluated. This choice is explained by the fact that these ensembles include fewer models than the top ones but perform no more than 0.2% worse according to Table 4 and Table 6. For each fold, the models included in the selected ensembles were re-trained and re-evaluated.

Table 7 shows the overall cross-validated performance of the right-lung models. The values to the left of the brackets are the mean values across folds, while the values in the brackets are their standard deviations. The cross-validated performance of the single best model (*sagcat*) is 0.4% higher than the non-cross-validated result (see Table 4), while the top performance of the ensemble dropped by 4.4%. Although the best ensemble previously showed better performance than any single model by a margin of 2.4%, after cross-validation, *sagcat* showed superior performance, incidentally also by a margin of 2.4%. Despite this, it cannot be concluded that ensembling is useless, since the ensemble still has higher Precision than the single best model, almost preserving the old margin (4.8% then vs. 4.2% now).

Table 8 demonstrates cross-validated by-fissure performance for the right-lung models. It can be seen that both before and after cross-validation, the models detected the horizontal fissures better than the oblique fissures. Although it was previously more evident with the ensemble, now the single best model performs similarly.

**Figure 6:**
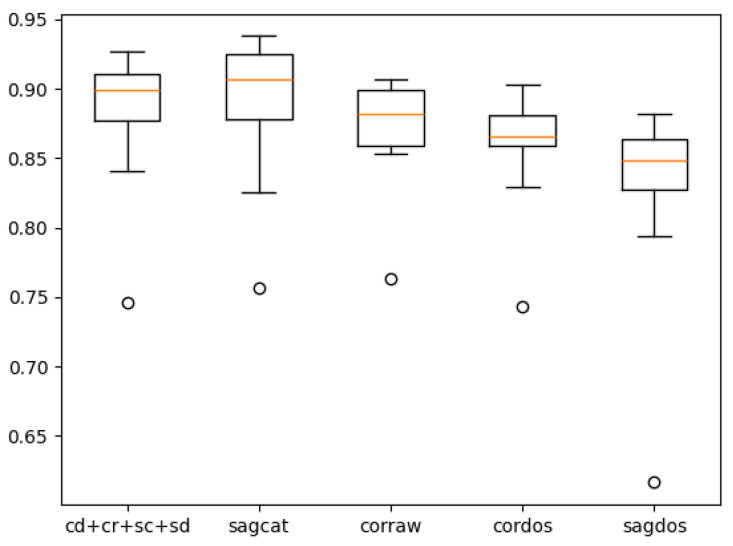
Plot for the right-lung models’ cross-validated performance on the oblique fissure.

**Figure 7:**
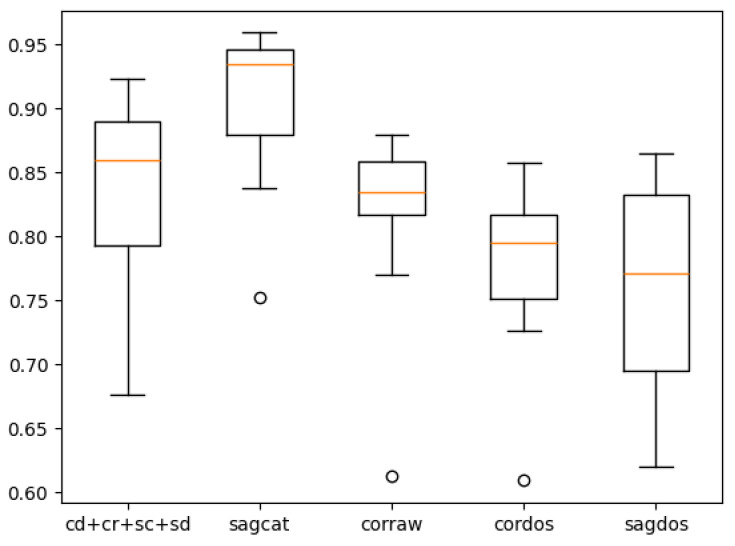
Plot for the right-lung models’ cross-validated performance on the horizontal fissure.

Figure 6 and Figure 7 contain box-with-whisker plots for the oblique and horizontal fissures. The box extends from the first quartile (Q1) to the third quartile (Q3) of the data, with a line at the median. The whiskers extend from the box to the farthest data point lying within 1.5× the inter-quartile range (IQR) from the box. Flier points are those past the ends of the whiskers.

**Figure 8:**
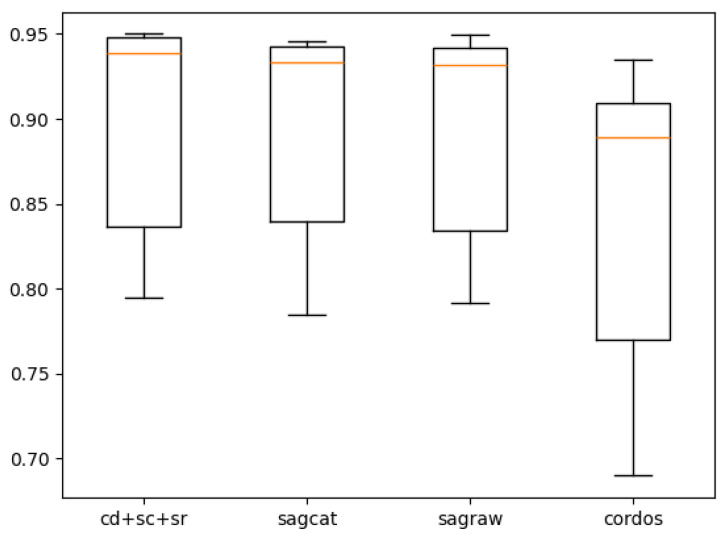
Plot for the left-lung models’ cross-validated performance.

Table 9 demonstrates cross-validated performance for the left-lung models. After cross-validation, the performance drops by 3.4%, but unlike the right-lung models, the ensemble still performs better, albeit by a smaller margin of 0.4% (compared to 0.7%). The boxplot for the left lung is shown in Figure 8.

### 3.3. Pulmonary Lobe Segmentation

Fissure masks can be used to obtain lung lobe masks by applying curvilinear approximation algorithms such as thin-plate splines [23]. Such lobe segmentation will not be accurate because not only fissures but also airways have a large influence on the appearance of lung lobes; for example, they occlude lung fissures’ visibility on a CT image in certain areas of the lung. Consequently, meaningful segmentation of the lobes requires either segmenting the airways [7] or segmenting the lobes directly [28]. Both of these approaches are beyond the scope of this paper.

Nevertheless, it is possible to perform an additional indirect assessment of the fissure segmentation quality using the lobe segmentation obtained using just the lung mask and fissure masks. For this purpose, the lobes found using ground-truth masks were compared with those found using predicted masks.

Before applying thin-plate splines, all components with sizes less than 500 voxels were removed from the right-lung fissure masks, and all components except the largest one were removed from the left-lung fissure mask. To eliminate misclassification, KNN was applied as described above. The lung mask was also used to establish the outer boundaries of the lobes.

The *cd + cr + sc + sd + sr* ensemble was used to segment the left-lung lobes, and the ensemble of all models (*cc + cd + cr + sc + sd + sr*) was used to segment the right-lung lobes, since these are the best ensembles for the left and right lung lobes, respectively. The *sagcat* model was also used to segment the lobes of both lungs. The comparison is shown in Table 10.

Here, UR, MR, LR, UL, and LL are the upper-right, middle-right, left-right, upper-left, and lower-left lobes, respectively; right avg, left avg, and all avg are the average scores for the right lung, left lung, and both lung lobes, respectively. In the first two rows, all avg is the average for the two ensembles. R or L in the index designates either the right or left lung, respectively, for the model.

**Figure 9:**
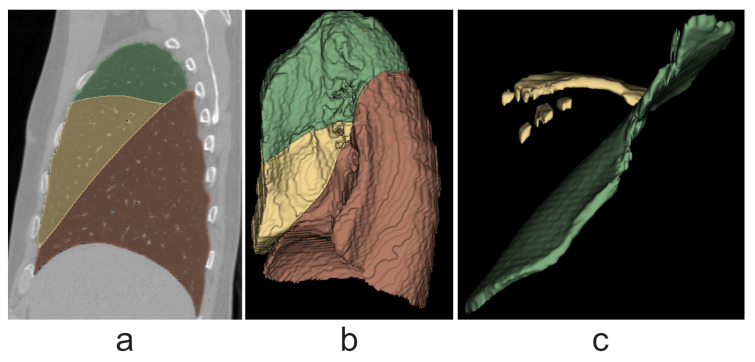
Right-lung lobes on one of the test images. (**a**) Lobes in the sagittal cross-section. Green, yellow and red colors are for the upper, middle, and red lobes, respectively. (**b**) A 3D model of lobes. (**c**) A 3D model of fissures. Green and yellow colors are for the oblique and horizontal fissures, respectively.

Ensembles of models perform better than any single model because they are less prone to type I errors. The results of left-lung lobe segmentation with ensembles are shown in Figure 9. Ensembles show a higher average result on both lungs, 0.989. In comparison, Wang et al. [28] achieved a median Dice score of 0.993, and Gerard et al. [25] achieved a median Dice score of 0.947.

**Figure 10:**
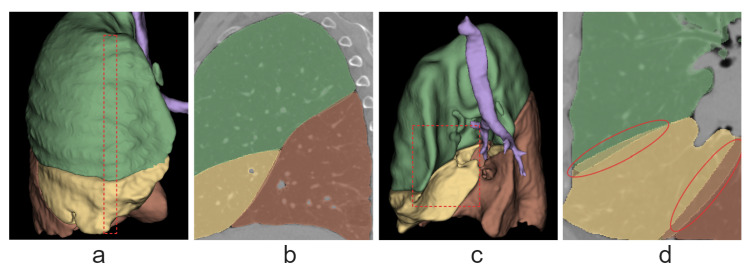
A comparison of true and predicted lobe masks of the right lung on one of the test images. Colors have the same meanings as in the Figure 9, except for purple, which denotes airways. Here, the values of $F_{1}$ are 0.992 for the upper lobe; 0.952 for the middle lobe; and 0.986 for the lower lobe. (**a**) A region on a 3D lobe model on which the section corresponding to (**b**) is highlighted with red. (**b**) A sagittal slice with both masks superimposed. (**c**) The 3D lobe model with masks superimposed in the area of the right lung adjacent to the mediastinum; the areas of discrepancy are circled in red. (**d**) The same area highlighted on the 3D model.

Figure 10 shows an example of a discrepancy between the predicted and true masks. The greatest discrepancy is observed in the regions of the lung adjacent to the mediastinum. In these regions, the fissures are the least segmented in both training and test samples as a result of various factors, such as other organs overlapping with the fissures.

## 4. Discussion

We have proposed a new method for pulmonary fissure segmentation on CT images based on the combination of DoS filtering, CNNs, and model ensembling techniques. Our method shows better results than the DoS method. Segmentation networks were trained on individual slices of sagittal and coronal cross-sections of preprocessed DoS-filtered CT images. Several models were also trained on slices of non-filtered images.

For the right lung, the best result is shown by the ensemble of models, with a modified F1 score of 0.916, compared to the standalone DoS method [6] score of 0.724. For the left lung, the ensemble of five models shows the best result, with a score of 0.933 (the standalone DoS method shows a score of 0.666).

While our method relies on 2D CNN models, it still accounts for volumetric information, firstly because a DoS filter is used as a preprocessing step and secondly since different components of the ensembles are trained on different cross-sections. Ensembles of networks trained on both coronal and sagittal slices give more accurate predictions than individual models or ensembles of models trained on only one of the cross-sections, which was confirmed experimentally. An axial cross-section was not used because the right horizontal fissure is almost perpendicular to the axis in most cases, making the data for training and evaluation very limited. Assuming that 32-bit floating point (FP) encoding is used, each model requires memory for at least one slice of 512×512 per inference pass, which is 1MB for a grayscale image and 3MB for an RGB image, which may be the original slice, a synthesized feature such as a DoS-filtered slice, or both. For comparison, Gerard et al. [24] used fixed-size image crops of 64×200×200, which is 5 MB for a 32-bit FP-encoded grayscale volume [24]. There is a trade-off between inference speed and memory consumption, as using very small batches or single samples slows down inference but reduces memory requirements.

Most modern computers have gigabytes of RAM. CPUs are able to segment fissures with CNN models by storing data and weights in the RAM rather than the video memory, although they will be much slower than GPUs. In addition to input and output data, there are model weights and intermediate outputs of hidden layers, the memory for which may not be reused in the same pass because the model can have skip connections. Assuming that segmenting the fissures in the scan will require no more than hundreds of times more memory than the input data themselves, the task can be accomplished on most modern computers, even with a 3D model.

The task of fissure segmentation is far from being completely solved and has many issues. Therefore, approaches that allow rapid experimentation are needed. In the inference and evaluation stage, the batch size only affects the execution time. In the training stage, the batch size is an important hyperparameter that has a huge impact on the learning process. For instance, a larger batch size gives a more accurate estimate of the true gradient, which makes the whole learning process more stable, especially at high learning rates. Two-dimensional CNNs give more freedom to choose the batch size because they are less constrained by memory resources. For this reason, approaches based on 2D CNNs, such as the proposed solution, have an advantage in research, even though this advantage is less significant in the inference process.

We performed cross-validation to ensure that the results are reliable, as the dataset was relatively small. Although on the right lung, the ensemble performed worse, according to the $F_{1}$ score, than the single model in cross-validation, it still had a higher Precision score, which is more important for applications such as lung lobe segmentation, as they are very sensitive to false positives. Models trained on data preprocessed with the DoS filter performed better both before and after cross-validation; therefore, the combination of the DoS filter and the CNN model gives more reliable prediction results than the CNN model trained on raw data.

The scope of the proposed method is limited to CT scans in which fissures are clearly visible. If a person cannot see fissures on a particular CT scan, they need additional data to do so, for example, a higher-resolution scan of the same patient taken under the same conditions. Patients rarely undergo CT scans twice in a row due to health risks. However, a dataset of high-resolution scans can be collected that can be algorithmically processed to mimic the performance of a low-resolution scanner. In this case, the segmentation masks for training and evaluation can be obtained from high-resolution scans, and the model can be trained and estimated on low-resolution scans. However, we then need a way to evaluate the accuracy of an algorithm that imitates low-resolution scanning.

In addition, in many cases, fissures remain partially invisible despite high scanning quality. Getting more people to annotate the same data and finding a consensus among them partially solves this problem, but the time and effort required increase proportionately. Another possible approach is to use other types of scanning, such as magnetic resonance imaging (MRI), along with CT. Although cases in which both types of imaging are performed on the same patient in a short period of time are rare, there are studies that address such cases [37,38].

Another weakness of our approach is that cases where the disease (more precisely, cancer or nodules mimicking cancer) obscures the lung fissures were rare in our dataset, while these are the most interesting cases, since one possible application of lobe detection is the diagnosis of lung cancer, which largely depends on whether the tumor has reached the inner boundaries of the lobe, which are defined by the lung fissures. Lobectomy, surgery to remove the diseased lobe of the lung, is more effective if the tumor does not cross the lobe boundary [5].

In addition, lung lobe segmentation was performed using fissure segmentation. Although the only purpose of lobe segmentation was to evaluate the quality of the fissure segmentation method, the lobe segmentation algorithm still shows results close to those of state-of-the-art methods, with an average Dice coefficient value of 0.989, which suggests the high efficiency of the proposed fissure segmentation method. In comparison, Wang et al. [28] achieved a median Dice score of 0.993, and Gerard et al. [25] achieved a median Dice score of 0.947.

To improve the fissure segmentation algorithm, we can collect more data, especially those where the appearance of fissures is affected by a disease, which will make the model more robust in edge cases. In addition, in future work, the performance of the proposed method and state-of-the-art 3D CNN models can be compared on the same data. Alternatively, future work can include the segmentation of airways and vessels to further improve the lobe segmentation algorithm. Also, the fissure segmentation algorithm can be used in the task of fissure integrity assessment.

## 5. Conclusions

We proposed a new method for pulmonary fissure segmentation on CT images using CNNs, image filtering, and model ensembles. The proposed method shows a modified F1 score of 0.916 for right-lung fissures and 0.933 for left-lung fissures, with a cross-validated performance of 0.894 and 0.899, respectively. We also developed a lung lobe segmentation method using the fissure segmentation method and thin-plate spline algorithm. It shows an average Dice coefficient of 0.989, so we conclude that the underlying fissure segmentation algorithm is effective. Future work may include making the fissure segmentation algorithm more robust in edge cases, particularly by utilizing a larger and more balanced dataset or developing an accurate lobe segmentation method. The fissure segmentation method can also be part of a fissure integrity assessment algorithm.

## Figures and Tables

**Table 1:** Abbreviation reference.

Model Name	Description	In-Ensemble Alias
*cordos*	A model trained on the slices retrieved in the coronal cross-section processed by the DoS filter.	*cd*
*corcat*	Coronal cross-section, combined processing.	*cc*
*corraw*	Coronal cross-section, without the DoS filter.	*cr*
*sagdos*	Sagittal cross-section, with the DoS filter.	*sd*
*sagcat*	Sagittal, combined.	*sc*
*sagraw*	Sagittal, without the DoS filter.	*sr*
*cd + cr + sc + sd*	An ensemble of the *cordos*, *corraw*, *sagcat*, and *sagdos* models.	-

**Table 2:** Testing models on the right lung.

Method	$F_{1}$	Precision	Recall	$F_{\mathrm{DoS}}$	$P_{\mathrm{DoS}}$	$R_{\mathrm{DoS}}$
Ensemble (all models)	0.683	0.571	0.878	0.914	0.898	0.943
*sagcat*	0.652	0.522	0.897	0.890	0.850	0.948
*sagraw*	0.620	0.482	0.907	0.876	0.828	0.945
*cordos*	0.653	0.542	0.855	0.875	0.833	0.939
*sagdos*	0.636	0.508	0.884	0.873	0.831	0.934
*corcat*	0.642	0.531	0.846	0.872	0.825	0.944
*corraw*	0.623	0.497	0.873	0.872	0.817	0.952

**Table 3:** DoS and ensemble comparison using the right lung.

Method	$F_{1}$	Precision	Recall	$F_{\mathrm{DoS}}$	$P_{\mathrm{DoS}}$	$R_{\mathrm{DoS}}$
Ensemble (all models)	0.687	0.572	0.884	0.916	0.898	0.946
Standalone DoS	0.305	0.733	0.201	0.724	0.763	0.708

**Table 4:** The five best ensembles for the right-lung fissure segmentation.

Method	$F_{1}$	Precision	Recall	$F_{\mathrm{DoS}}$	$P_{\mathrm{DoS}}$	$R_{\mathrm{DoS}}$
All 6 models	0.683	0.571	0.878	0.914	0.898	0.943
*cd + cr + sc + sd*	0.686	0.581	0.867	0.914	0.901	0.938
*cc + cr + sc + sd*	0.684	0.578	0.867	0.913	0.900	0.938
*cd + cr + sc + sr*	0.680	0.570	0.873	0.913	0.899	0.939
*cc + cd + sc + sr*	0.689	0.587	0.860	0.913	0.903	0.934

**Table 5:** $F_{1}$ and $F_{DoS}$ scores depending on right-lung fissure and model.

Method	$F_{1}^{\mathrm{Oblique}}$	$F_{1}^{\mathrm{Horizontal}}$	$F_{\mathrm{DoS}}^{\mathrm{Oblique}}$	$F_{\mathrm{DoS}}^{\mathrm{Horizontal}}$
*cc + cd + cr + sc + sd + sr*	0.678	0.682	0.909	0.915
*sagcat*	0.643	0.653	0.884	0.887
*sagraw*	0.615	0.617	0.873	0.867
*cordos*	0.652	0.638	0.877	0.856
*corraw*	0.630	0.590	0.873	0.855
*sagdos*	0.630	0.632	0.871	0.855
*corcat*	0.652	0.608	0.880	0.844

**Table 6:** Our four best ensembles, the best model, and the DoS method on the left lung. *sagcat R* and *sc+sd+sr R* are the best model and the best ensemble trained on the right-lung slices.

Method	$F_{1}$	Precision	Recall	$F_{\mathrm{DoS}}$	$P_{\mathrm{DoS}}$	$R_{\mathrm{DoS}}$
*sagcat*	0.725	0.753	0.724	0.926	0.933	0.922
*cd + cr + sc + sd + sr*	0.737	0.745	0.747	0.933	0.944	0.923
*cd + sc + sr*	0.738	0.743	0.753	0.932	0.940	0.927
*sagcat R*	0.721	0.658	0.826	0.907	0.933	0.886
*sc + sd + sr R*	0.710	0.626	0.846	0.907	0.933	0.886
Standalone DoS	0.285	0.702	0.188	0.666	0.767	0.617

**Table 7:** Right-lung models’ overall cross-validated performance.

Method	$F_{1}$	Precision	Recall	$F_{\mathrm{DoS}}$	$P_{\mathrm{DoS}}$	$R_{\mathrm{DoS}}$
*cd + cr + sc + sd*	0.550 (0.051)	0.452 (0.053)	0.786 (0.064)	0.870 (0.055)	0.900 (0.048)	0.858 (0.061)
sagcat	0.559 (0.059)	0.425 (0.076)	0.904 (0.068)	0.894 (0.055)	0.858 (0.057)	0.942 (0.058)
*corraw*	0.522 (0.049)	0.394 (0.047)	0.833 (0.053)	0.861 (0.047)	0.824 (0.049)	0.912 (0.046)
*cordos*	0.507 (0.045)	0.399 (0.050)	0.768 (0.058)	0.839 (0.048)	0.828 (0.053)	0.865 (0.051)
*sagdos*	0.495 (0.060)	0.384 (0.062)	0.781 (0.071)	0.810 (0.073)	0.819 (0.085)	0.823 (0.073)

**Table 8:** Right-lung models’ by-fissure performance with 10-fold cross-validation.

Method	$F_{1}^{\mathrm{Oblique}}$	$F_{1}^{\mathrm{Horizontal}}$	$F_{\mathrm{DoS}}^{\mathrm{Oblique}}$	$F_{\mathrm{DoS}}^{\mathrm{Horizontal}}$
*cd + cr + sc + sd*	0.541 (0.050)	0.565 (0.061)	0.881 (0.051)	0.839 (0.072)
*sagcat*	0.529 (0.066)	0.622 (0.051)	0.887 (0.054)	0.903 (0.062)
*corraw*	0.518 (0.045)	0.517 (0.064)	0.871 (0.040)	0.816 (0.074)
*cordos*	0.510 (0.045)	0.482 (0.056)	0.857 (0.042)	0.774 (0.067)
*sagdos*	0.486 (0.063)	0.506 (0.057)	0.825 (0.074)	0.761 (0.080)

**Table 9:** Left-lung models’ performance with 10-fold cross-validation.

Method	$F_{1}$	Precision	Recall	$F_{\mathrm{DoS}}$	$P_{\mathrm{DoS}}$	$R_{\mathrm{DoS}}$
*cd + sc + sr*	0.726 (0.061)	0.787 (0.067)	0.690 (0.056)	0.899 (0.065)	0.950 (0.064)	0.869 (0.065)
*sagcat*	0.707 (0.059)	0.782 (0.064)	0.664 (0.056)	0.895 (0.065)	0.945 (0.054)	0.869 (0.065)
*sagraw*	0.722 (0.059)	0.738 (0.072)	0.722 (0.057)	0.894 (0.062)	0.926 (0.066)	0.873 (0.058)
*cordos*	0.672 (0.072)	0.752 (0.063)	0.621 (0.079)	0.847 (0.085)	0.929 (0.063)	0.794 (0.099)

**Table 10:** The Dice scores of the results of lobe segmentation by two ensembles and the *sagcat* model with the approximation algorithm applied. UR, MR, LR, UL, and LL stand for upper right, middle right, lower right, upper left, and lower left, respectively.

Method	UR	MR	LR	Right avg	UL	LL	Left avg	All avg
All-models ensemble *R*	0.991	0.977	0.991	0.986	-	-	-	0.989
*cd + cr + sc + sd + sr L*	-	-	-	-	0.993	0.993	0.993	0.989
*sagcat L*	0.980	0.952	0.986	0.973	0.994	0.994	0.994	0.981

## Data Availability

The imaging data that were used in this study can be shared upon request.
